# Metabolic pathways from the gut metatranscriptome are associated with COPD and respiratory function in lung cancer patients

**DOI:** 10.3389/fcimb.2024.1381170

**Published:** 2024-11-20

**Authors:** David Dora, Peter Revisnyei, Anna Mihucz, Peter Kiraly, György Szklenarik, Edit Dulka, Gabriella Galffy, Zoltan Lohinai

**Affiliations:** ^1^ Department of Anatomy, Histology, and Embryology, Semmelweis University, Budapest, Hungary; ^2^ Department of Telecommunications and Media Informatics, Budapest University of Technology and Economics, Budapest, Hungary; ^3^ County Hospital of Torokbalint, Torokbalint, Hungary; ^4^ Translational Medicine Institute, Semmelweis University, Budapest, Hungary

**Keywords:** gut microbiome, metabolic pathways, PFAMs, COPD, lung function, metagenome, metatranscriptome

## Abstract

**Introduction:**

Changes in the human gut microbiome have been linked to various chronic diseases, including chronic obstructive pulmonary disease (COPD). While substantial knowledge is available on the genomic features of fecal communities, little is known about the microbiome’s transcriptional activity. Here, we analyzed the metatranscriptomic (MTR) abundance of MetaCyc pathways, SuperPathways, and protein domain families (PFAM) represented by the gut microbiome in a cohort of non-small cell lung cancer (NSCLC) patients with- or without COPD comorbidity.

**Methods:**

Fecal samples of 40 NSCLC patients with- or without COPD comorbidity were collected at the time of diagnosis. Data was preprocessed using the Metaphlan3/Humann3 pipeline and BioCyc^©^ to identify metabolic SuperPathways. LEfSe analysis was conducted on Pathway- and PFAM abundance data to determine COPD- and non-COPD-related clusters.

**Results:**

Key genera *Streptococcus, Escherichia, Gemella*, and *Lactobacillus* were significantly more active transcriptionally compared to their metagenomic presence. LEfSe analysis identified 11 MetaCyc pathways that were significantly overrepresented in patients with- and without COPD comorbidity. According to Spearman’s rank correlation, Smoking PY showed a significant negative correlation with *Glycolysis IV*, *Purine Ribonucleoside Degradation* and *Glycogen Biosynthesis I*, and a significant positive correlation with *Superpathway of Ac-CoA Biosynthesis* and *Glyoxylate cycle*, whereas forced expiratory volume in the first second (FEV1) showed a significant negative correlation with *Glycolysis IV* and a significant positive correlation with *Glycogen Biosynthesis I*. Furthermore, COPD patients showed a significantly increased MTR abundance in ~60% of SuperPathways, indicating a universally increased MTR activity in this condition. FEV1 showed a significant correlation with *SuperPathways Carbohydrate degradation*, *Glycan biosynthesis*, and *Glycolysis*. Taxonomic analysis suggested a more prominent MTR activity from multiple *Streptococcus* species, *Enterococcus (E.) faecalis, E. faecium* and *Escherichia (E.) coli* than expected from their metagenomic abundance. Multiple protein domain families (PFAMs) were identified as more associated with COPD, *E. faecium, E.coli*, and *Streptococcus salivarius*, contributing the most to these PFAMs.

**Conclusion:**

Metatranscriptome analysis identified COPD-related subsets of lung cancer with potential therapeutic relevance.

## Introduction

Changes in the human gut microbiome have been linked to a variety of chronic diseases, including obesity, diabetes, inflammatory bowel disease (IBD), cancer, and cardiovascular disease (Shreiner et al., 2015). Although there is a substantial body of knowledge on the metagenomic features of fecal communities (Human Microbiome Project Consortium, 2012; Le Chatelier et al., 2013), little is known about the microbiome’s transcriptional activity. The linkage between the genotype and phenotype of the commensal gut flora might be explained by understanding their metatranscriptome, which represents the functional ecology of the human gut microbiome (Franzosa et al., 2014; Abu-Ali et al., 2018). In a clinical setting, the unequivocal role of the gut metatranscriptome and its distinction from metagenomics was already shown in IBD (Schirmer et al., 2018), in metastatic melanoma (Peters et al., 2019,) and murine inflammation models (Jovel et al., 2022).

Chronic Obstructive Pulmonary Disease (COPD) represents an enormous public health burden with an age-standardized incidence of 1.46% in high-SDI- and 1.02% in middle-to-low-SDI countries. The estimated prevalence of COPD is almost 10% in the 30-79 year-old population, being responsible for 3.197 million deaths every year worldwide (Wang et al., 2022; Li et al., 2023). In lung cancer patients, COPD comorbidity poses an additional risk, with significantly decreased overall survival, particularly in the case of squamous cell carcinoma (Wang et al., 2018; Yi et al., 2018). COPD may have a role in lung cancer development by increasing oxidative stress and associated DNA damage, persistent exposure to pro-inflammatory cytokines, inhibition of DNA repair systems, and enhanced cellular proliferation (Papi et al., 2004; Barnes and Adcock, 2011; Durham and Adcock, 2015; Young et al., 2015).

Both COPD and lung cancer were recently associated with dysbiotic airway microbiota and commonly occur alongside gastrointestinal (GI) disorders, possibly through the gut-lung axis (Bingula et al., 2017; Dang and Marsland, 2019; Enaud et al., 2020; Bulanda and Wypych, 2022; Qu et al., 2022). Comprehensive metagenomic sequencing of the gut microbiome provided valuable insights into differentially expressing taxa and changes in the metabolome between healthy and COPD patients (Bowerman et al., 2020; Li N. et al., 2021), and correlation with respiratory function (Marfil-Sánchez et al., 2021). Recently, gut dysbiosis was also associated with the frequency of viral pulmonary infections and declining lung function in COPD patients (Sencio et al., 2021; Chiu et al., 2022). In addition, others showed that the lipopolysaccharide component of commensal bacterium *Parabacteroides goldsteinii* might have a pivotal role in COPD pathogenesis (Lai et al., 2022). Despite multiple lines of evidence of intricate gut-lung crosstalk possibly mediated by the microbiome, these studies are based on genomic sequencing and provide no insights into related metatranscriptomics.

In the present study, we compared the Metagenomic (MG) and Metatranscriptomic (MTR) abundance of bacterial species and genera using fecal samples of 40 non-small cell lung cancer (NSCLC) patients. Furthermore, we classified patients according to COPD comorbidity (COPD vs non-COPD) and revealed emerging metabolic pathways the gut microbiome represents. We also aimed to analyze the taxonomic representation of pathways and protein domain families (PFAMs) and correlate them with essential respiratory function parameters, such as Smoking, CAT score, or FEV1. This is the first study to compare gut metatranscriptomic signatures according to COPD status and respiratory function.

## Materials and methods

### Study population

A total of 40 patients diagnosed with NSCLC and receiving standard-of-care therapy approved by the Institutional Oncology Team were enrolled in our study cohort between 2019 and 2021 at the County Hospital of Pulmonology, Torokbalint, Hungary. **Table 1** shows the clinical parameters of the study cohort, where patients were classified as non-COPD and COPD. Clinicopathological data included age, gender, smoking pack year (PY), body mass index (BMI, kg/m^2^), and COPD Global Initiative for Chronic Obstructive Lung Disease (GOLD) stadium at the time of lung cancer diagnosis. Patients underwent COPD Assessment Test (CAT) to determine their CAT score and measurement of Forced Expiratory Volume in 1 second (FEV1) within one week of obtaining fecal samples. All patients were assessed with Eastern Cooperative Oncology Group (ECOG) 0-1 performance status at the time of fecal sampling. Before sampling, all COPD patients received standard-of-care therapy according to the GOLD guidelines. Patients receiving systemic antibiotic therapy or having acute exacerbation within three months of fecal sampling were not included in the study cohort.

**Table 1:** Clinicopathological characteristics of the patient cohort.

	Non-COPD N=16	COPD N=21	p-value
Age [years (mean)]	62.13 (± 11.6)	60.9 (± 8.1)	0.103
Gender male female	8 (50%) 8 (50%)	9 (43%) 12 (57%)	0.746
Smokin PY (mean)	30.2 (± 12.3)	56.6 (± 27.4)	0.012*
Body mass index (BMI) >30 kg/m^2^ ≤30 kg/m^2^ N/A	5 (31%) 10 (63%) 1 (6%)	5 (24%) 13 (62%) 3 (14%)	>0.999
COPD Gold stadium GOLD 1 GOLD 2 GOLD 3 GOLD 4	N/A	2 (10%) 4 (20%) 12 (57%) 3 (13%)	
CAT score (mean)	6.8 (± 5.5)	12.1 (± 6.7)	0.049*
FEV1%	91.9 (± 11.5)	61.2 (± 15.3)	<0.001***

Statistical significance *P < 0.05; ***P<.001, all p-values were two-sided.

N/A means data not available.

### Sample processing

Patients were enrolled in the study after signing informed consent and providing baseline stool samples collected within seven days of diagnosis. All samples were stored in the -80°C freezer on the same collection day until sequencing. The fecal samples were processed as previously described (Dora et al., 2023b), in brief: 300 ml of cool 80% aqueous methanol was added to homogenizer tubes for every 100 mg of sample. The sample preparation procedures were carried out on dry ice with cooled instruments. The Bead Ruptor 24 Elite (OMNI International) with the Heart program (6 m/s, 30 s) was used to homogenize the samples. The samples were then vortexed for 10 seconds before being centrifuged for 10 minutes at 13,000 rpm and 4°C. The supernatant was collected in a 96-well filter plate and centrifuged for 5 minutes at 4°C at 700 g.

### Shotgun metagenomic pipeline

We used 100 mg stool samples in ZR Bashing Bead Lysis Tubes with ZymoBIOMICS 96 MagBead DNA kit for whole DNA extraction. We used continuous bead beating for 40 minutes and centrifuged the lysate for 1 min at 10,000 x g. 200 μl supernatant was mixed with 25 μl ZymoBIOMICS™ MagBinding Beads, then shaked for 10 minutes. After placing the tubes on a magnetic rack and removing the supernatant, 500 μl ZymoBIOMICS™ MagBinding Buffer was added to each sample and mixed for 1 minute. The beads were pelleted and washed two times with 500 μl of ZymoBIOMICS™ MagWash 1 and 900 μl ZymoBIOMICS™ MagWash 2, respectively, for 1 min. The beads were dried at 55°C for 10 min. Then eluted in 50 μl RNAse/DNAse free water. The DNA concentration was measured with a Qubit fluorimeter.

From each sample, 65 ng was used as input for library preparation by KAPA HyperPlus kit as per the manufacturer instructions, with size selection for ~200bp peak fragment size (TapeStation 2200, High Sensitivity D1000 ScreenTape^®^). The samples were sequenced on the NextSeq500 platform, 2x150bp, with ~10M read pairs.

### Shotgun metatranscriptomic pipeline

Quick RNA Fecal/Soil Microbe Microprep kit (Zymo Research) was used for RNA extraction, starting with 40 minutes of continuous bead beating of 100mg stool sample with 1mL of S/F RNA Lysis Buffer added. After centrifuging for 1 minute, 400 μl of supernatant was filtered through (3000 x g, 30 seconds) in Zymo-Spin™ IIICG Column2, mixed with 95% ethanol in a 1:1 ratio, and transferred to a new Zymo-Spin™ IIICG Column2 for RNA binding. The column was washed with 400 μl RNA Prep Buffer, then the RNA was eluted in 100 μl Nuclease-free water, and transferred to a prepared Zymo-Spin™ III-HRC Filter to be centrifuged at 8000 x g for 3 minutes. The filtered RNA was mixed with 200 μl RNA binding buffer and an equal volume of 95% ethanol. The mixture was loaded on Zymo-Spin™ IC Column2 and washed with RNA wash buffer for DNAse I treatment (5 μl DNAse I, 35 μl DNA digestion buffer, incubation for 15 minutes) after the supernatant was discarded. The treated RNA was washed in 400 μl prep buffer 1x and RNA wash buffer 2x, then eluted in 15 μl RNAse/DNAse free water. The isolated RNA’s concentration and integrity were verified with a Qubit fluorometer (Qubit HS RNA kit, Thermofisher) and Labchip GX Touch, RNA Pico Sensitivity Assay (Perkin Elmer). For ribosomal depletion of RNA samples, 250 ng input was used with NEBNext rRNA depletion kit v2 (human/mouse/rat) and NEBNext rRNA depletion kit (bacteria) hybridization probes (probes mixed with a ratio of 1:1) following the manufacturer’s instructions, followed by library preparation using Nextflex Rapid Directional RNA-Seq kit, following the manufacturer’s instruction, with 12 min of fragmentation for a target library size of 320-430bp.

KAPA Single indexes for Illumina were used for indexing with 10 PCR cycles in the library preparation procedure. The final library concentration and size were evaluated with a Qubit fluorometer, Labchip GX Touch, and DNA NGS 3k assay. The samples were sequenced on the NextSeq platform, 2x81bp, with ~20M read pairs.

### Quality check

The adaptor-trimmed reads were quality-filtered to ensure a minimum mean Q-score of 30. Quality checks were performed using fast QC (Andrews, 2010), including removing adapter regions, low-quality reads, and human DNA contaminations. This process involved passing per sequence quality score, per base N content, and adapter content assessments as outlined in bwa (version 0.7.4-r385) (Andrews, 2010). The forward and reverse reads were concatenated as recommended by the authors for the analysis with Humann3 (version v3.0.0.alpha.4) (Beghini et al., 2021) using the CHOCOPhlAn_201901 database, and the EC-filtered uniref90_201901 database for translated search. SortMeRNA (Kopylova et al., 2012) was used to remove rRNA sequences in MTR data.

### Pathway analysis of shotgun metagenomic and metatranscriptomic data

The results consisted of tables with raw read per kilobase (RPK) values for each record and the path abundance table, with the calculated raw pathway abundance (expressed as the function of the abundance of reactions constituting the Pathway, which is calculated as the sum of over the abundance of genes involved). Reads not mapped to either feature in the databases were counted under the label “UNMAPPED.” Similarly, mapped reads that could not be integrated into any pathways were assigned as “UNINTEGRATED.” Each Pathway is also stratified by taxonomy, labeled “unclassified” if no taxonomy can be inferred. For comparison of samples, the RPK values were normalized to copies per million (CPM) with the human_renorm_table script; then, reactions were regrouped with the humann_regroup_table script. Records not regrouped to the new features appeared as “UNGROUPED.” For each Pathway, diversity indexes (Shannon and Simpson) were calculated using the species data with the R package vegan (https://CRAN.R-project.org/package=vegan).

MetaCyc pathways were included in further analyses if their populational abundance (in the whole cohort) reached at least 0.1%. Plus, only pathways present in at least 25% of the whole cohort population were included, leaving 124 metabolic pathways from a total of 556. For ease of interpretation and comparison between samples, pathways were grouped into superclasses according to the Metacyc hierarchy (SuperPathways, BioCyc^©^) (Karp et al., 2019), where a total of 61 SuperPathways were identified. SuperPathways contributing to at least 1% of total abundance were included in further analysis (n=17). The normalization of abundance values was done with central log ratio (clr) transformation in R (https://rdrr.io/github/thomazbastiaanssen/Volatility/man/clr_lite.html). The values were transformed in several ways according to the possible methods of the clr_lite function.

### Assessment of PFAMs

The high-quality metatranscriptomic reads were assembled into contigs using MetaSPAdes (Nurk et al., 2017). After assembly, gene prediction was conducted on the contigs using Prodigal (Hyatt et al., 2010), and the predicted gene sequences were translated into protein sequences for further analysis. The identification of protein domain families was carried out using the Pfam database, accessed at http://pfam.xfam.org/ (Mistry et al., 2021). This involves scanning the translated protein sequences against the Pfam-A database with HMMER, a tool available at http://hmmer.org/. The Pfam-A database comprises a comprehensive collection of protein domain families represented as profile hidden Markov models (HMMs). HMMER settings were adjusted to balance sensitivity and specificity, employing the default settings for initial scans. Pfams contributed to at least 0.1% of total abundance (n=201), with more-than-zero abundance present in at least 25% of patients included.

### Linear discriminant analysis effect size

Linear discriminant analysis effect size (LEfSe) (Segata et al., 2011) was conducted on CLR-normalized BioCyc^©^ Pathway and protein family (Pfam) abundance data to determine Pathway- and Pfam clusters that exhibit significant differences in occurrence between patients with and without COPD. LEfSe analysis was performed using the Galaxy computational tool (http://huttenhower.sph.harvard.edu/galaxy/) (Galaxy Community, 2022) to estimate the effect size of each differentially abundant feature, with a threshold on LDA scores set at 2.0 and alpha values at 0.01.

### Statistical analyses

First, the Shapiro-Wilks test was used to determine if data is normally distributed. Differential abundance testing of Metacyc Superpathways and diversity comparisons were done using the Wilcoxon rank-sum test. The associations between the relative abundances of taxa and clinical parameters were investigated with Spearman’s rank correlation, P-values less than 0.05 indicate the significance, and all p-values were two-sided.

Hierarchical cluster analysis was conducted on the dataset using Python. Key Python libraries, including Pandas (https://pandas.pydata.org/docs/whatsnew/index.html), Seaborn (https://seaborn.pydata.org/whatsnew/index.html), Matplotlib (https://matplotlib.org/stable/project/citing.html), and SciPy (Virtanen et al., 2020), were utilized for data handling and visualization. The dataset underwent preprocessing to ensure compatibility with clustering analysis, transforming abundances to Z-scores. SciPy’s linkage method was employed for hierarchical clustering with a complete linkage method. This was followed by dendrogram generation using *SciPy*, assisting in visualizing clustering hierarchy and cluster determination. A heatmap was then created with Seaborn, integrating the clustering results by reordering data according to the hierarchical structure.

## Results

**Figure 1:**
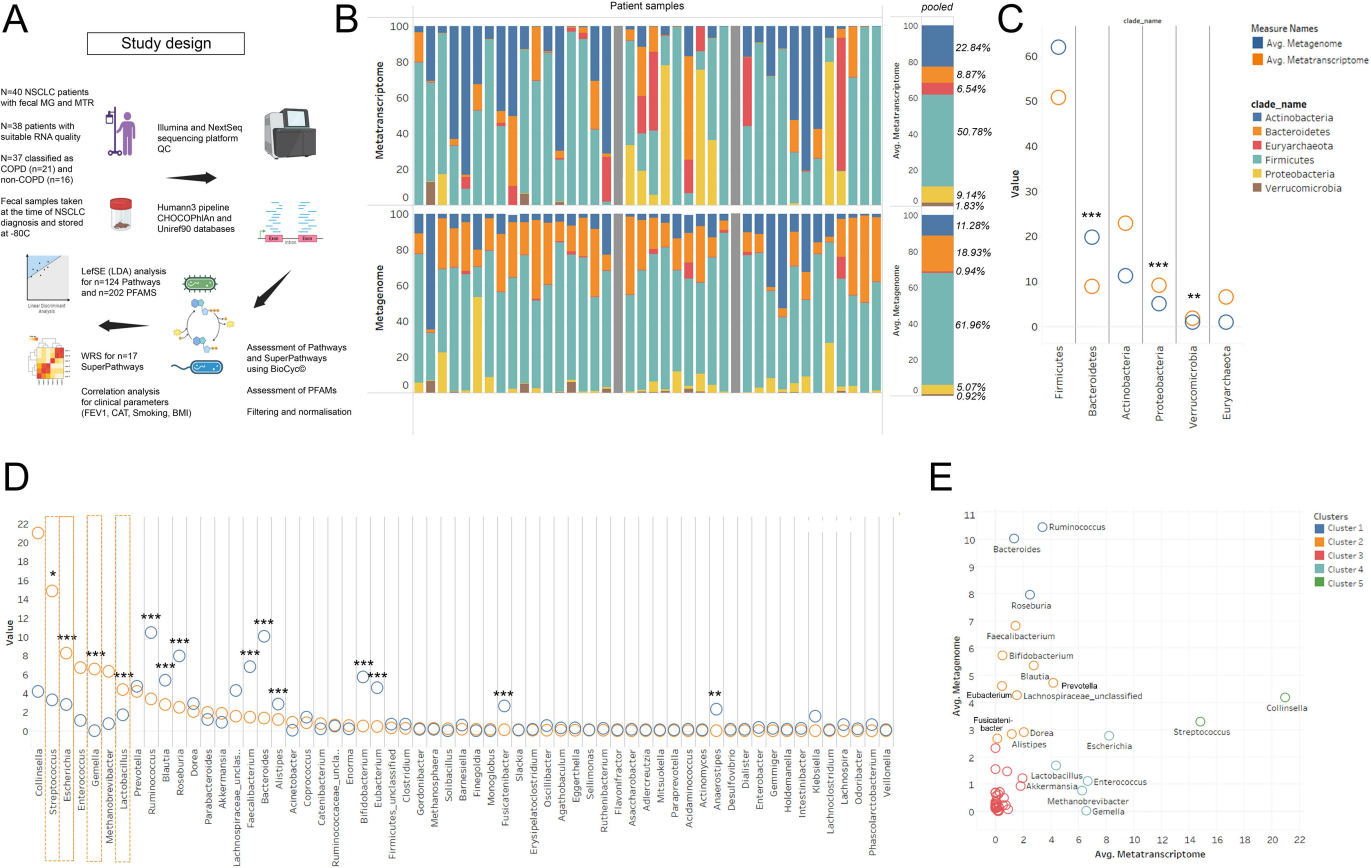
MG vs MTR according to phyla and genera. Study design and research workflow is shown in panel **(A)** 100% stacked bar chart shows Metatranscriptomic (MTR) and Metagenomic (MG) signatures at phylum level evaluated in 40 patients (grey bar: did not pass QC), **(B)**. *Bacteroidetes* showed significantly higher MG than MTR abundance (p<0.001), while *Proteobacteria* and *Verrucomicrobia* were more represented at the MTR level (p<0.001). *Actinobacteria* and *Euryarcheota* indicated increased MTR abundance, though not statistically significant **(C)**. At the genus level, *Ruminococcus, Blautia, Roseburia, Faecalibacterium, Bacteroides, Alistipes, Bifidobacterium, Eubacterium, Fusicatenibacter* (p<0.001 for all) and *Anaerostipes* (p=0.003) had higher MG than MTR abundance, suggesting lower transcriptomic activity **(D)**. Conversely, genera like *Collinsella, Streptococcus, Enterococcus, Gemella, Methanobrevibacter, Escherichia*, and *Lactobacillus* exhibited higher MTR than MG abundance, with *Streptococcus* (S), *Escherichia, Gemella*, and *Lactobacillus* being statistically significant (p<0.031 for S., p<0.001 for others) (**D**, orange dashed line). XY chart analysis **(E)** clustered genera based on their MG and MTR abundances into five distinct groups, varying from very high MG but low MTR (cluster 1, blue) to low MG but very high MTR (cluster 5, green). Statistical significance *P < 0.05; **P < 0.01, ***P<.001, all p-values were two-sided.

A total of 40 advanced stage (stage IIIB/IV) NSCLC patients who underwent fecal metagenomic (MG) and metatranscriptomic (MTR) sequencing were included in our study. 38 patients had their metatranscriptomic sequencing data pass the quality check. 2 patients were excluded due to low-quality RNA yields. 16 patients were categorized as non-COPD, and 21 patients were categorized as COPD. One patient had no relevant clinical data concerning COPD comorbidity. Patient clinicopathological data included age, gender, COPD GOLD stadium, CAT score, FEV1, smoking pack year (PY), and BMI (**Table 1**). The median age of the study cohort was 61.3 years [95% CI: 58.2 to 65.1]. The study design is shown in **Figure 1A**.

### Metagenomic vs metatranscriptomic abundance in the context of taxonomy

First, we aimed to assess the MTR and corresponding MG signatures of major bacterial taxa at the species and genus level in the whole cohort, irrespective of COPD status, to reveal taxonomic units with coherent and contrasting MG and MTR abundances. Bacterial phylum distribution according to MG and MTR is shown in **Figure 1B**. Statistically, *Bacteroidete*s abundance is significantly higher at the MG level (compared to MTR, p<0.001), whereas *Proteobacteria* and *Verrucomicrobia* are significantly stronger represented at the MTR level (compared to MG, p<0.001). There was a trend towards increased MTR abundance in the case of *Actinobacteria* (p=0.057) and *Euryarcheota* (p=0.075), but statistically not significant due to high standard deviations (**Figure 1C**).

Among genera, a series of bacteria showed significantly higher MG representation than MTR, including *Ruminococcus* (p<0.001), Blautia (p<0.001), *Roseburia* (p<0.001), *Faecalibacterium* (p<0.001), *Bacteroides* (p<0.001), *Alistipes* (p<0.001), *Bifidobacterium* (p<0.001), *Eubacterium* (p<0.001), *Fusicatenibacter* (p<0.001) and *Anaerostipes* (p=0.003), indicating that these genera are not as transcriptionally active, as their DNA abundance suggests (**Figure 1D**). In contrast, *Collinsella, Streptococcus, Escherichia, Enterococcus, Gemella, Methanobrevibacter*, and *Lactobacillus* showed a higher MTR abundance than expected based on their MG abundance, with statistically significant differences in the case of *Streptococcus* (p=0.031), *Escherichia* (p<0.001), *Gemella* (p<0.001) and *Lactobacillus* (p<0.001) (**Figure 1D**, orange dashed line). **Figure 1E** shows the same analysis in a scatter chart, where clusters represent genera with very high MG, but low MTR (cluster 1, blue), high MG, but low MTR (cluster 2, orange), both low MG and MTR (cluster 3, red), low MG, but high MTR (cluster 4, light blue), and low MG, but very high MTR (cluster 5, green).

### Metabolic pathways overrepresented in COPD and non-COPD patients

**Figure 2:**
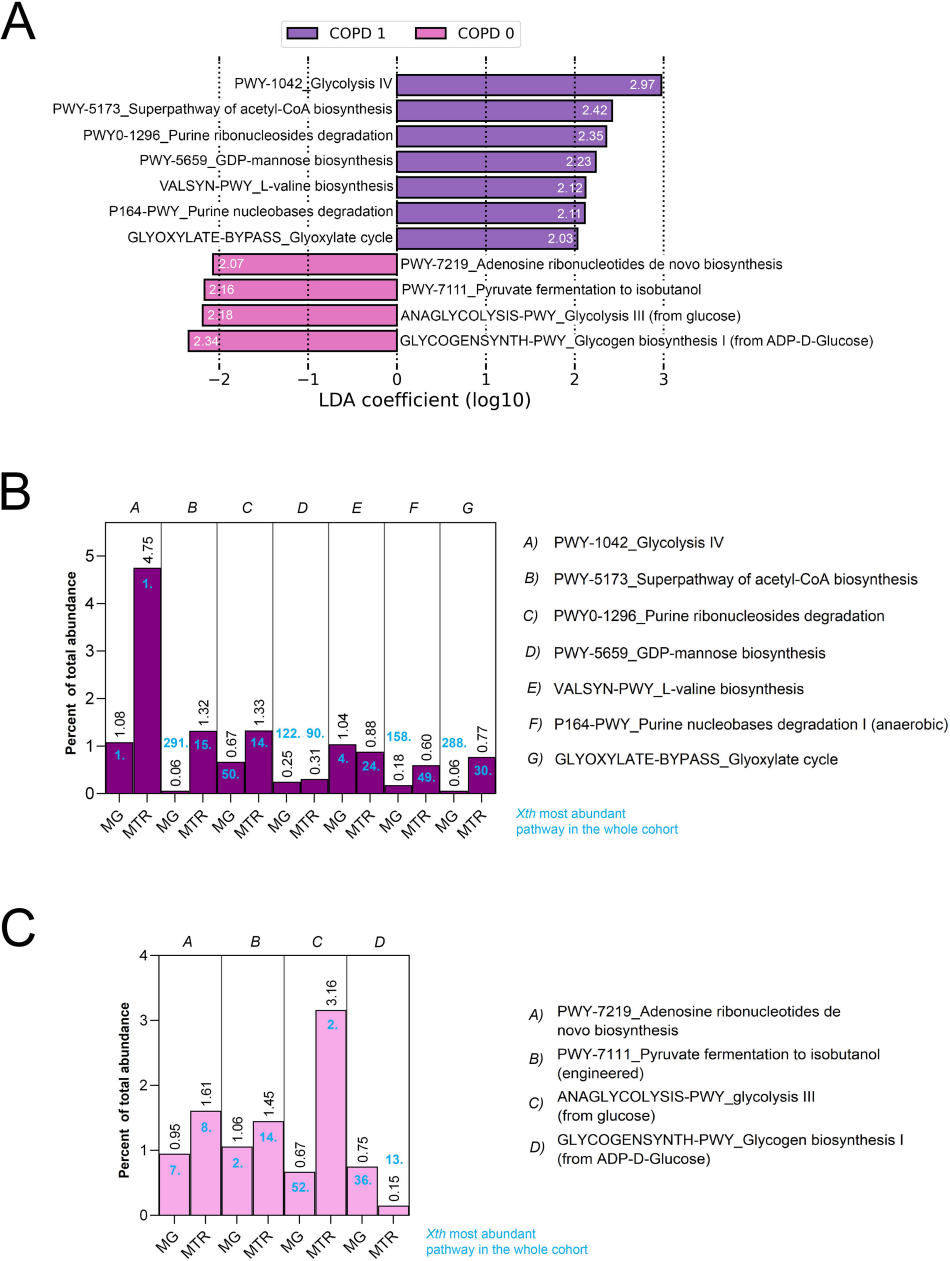
LEfSe analysis and contribution of key pathways to the metatranscriptome. Linear Discriminant Analysis Effect Size (LEfSe) identified 11 key metabolic pathways significantly discriminating between COPD and non-COPD patients based on MTR abundance (FDR<0.05, _Log10_LDAcoeff>2). _Log10_LDAcoeff values are displayed on horizontal bars showing pathways **(A)**. Of these, 7 pathways, including *Glycolysis IV* (LDAcoeff=2.97), *Superpathway of Acetyl-CoA biosynthesis* (LDA=2.42), *Purine ribonucleosides degradation* (LDAcoeff=2.35), *GDP-mannose biosynthesis* (LDAcoeff=2.23), *L-valine biosynthesis* (LDAcoeff=2.12), *Purine nucleobases degradation* (LDAcoeff=2.11), and *Glyoxylate cycle* (LDAcoeff=2.03) were overrepresented in COPD patients, while 4 pathways, such as *Adenosine ribonucleotides de novo biosynthesis* [LDAcoeff=(2.07)], *Pyruvate fermentation to isobutanol* [LDAcoeff=(2.16)], *Glycolysis II* [LDAcoeff=(2.18)], and *Glycogen biosynthesis I* [LDAcoeff=(2.34)] were prevalent in non-COPD patients **(A)**. Further analysis revealed that in the microbial metabolism of the gut, *Glycolysis IV* and *III* exhibited the highest (4.75% and 3.16%, respectively), while *GDP-mannose biosynthesis* (0.31%) *and Glycogen biosynthesis I* (0.15%) was the lowest MTR abundance among the key pathways **(B, C)**.

First, we performed Linear Discriminant Analysis Effect Size (LEfSe) to determine pathways having the most remarkable effect size discriminating COPD vs. Non-COPD patients regarding their metatranscriptomic abundance (key pathways). A total of 11 pathways showed statistically significant (FDR<0.05) and considerable (_Log10_LDAcoeff>2) discriminating power between the COPD and non-COPD populations. 7 of these pathways showed greater effect size towards the COPD-phenotype and 4 pathways towards the non-COPD phenotype (**Figure 2A**). Metabolic pathways (MetaCyc^©^) *Glycolysis IV, Superpathway of Acetyl-CoA biosynthesis, Purine ribonucleosides biodegradation, GDP-mannose biosynthesis, L-valine biosynthesis, Purine nucleobases degradation* and *Glyoxylate cycle* were overrepresented in patients with COPD comorbidity, whereas metabolic pathways *Adenosine ribonucleotides de novo biosynthesis, Pyruvate fermentation to isobutanol, Glycolysis III (from glucose)* and *Glycogen biosynthesis I (from ADP-D-glucose)* were overrepresented in patients without COPD comorbidity.

To determine whether these key pathways represent a considerable abundance in the microbial metabolism of the gut, we assessed their contribution to the total metagenomic (MG) and metatranscriptomic (MTR) abundance in percentage and their position among the most abundant pathways (**Figures 2B, C**). Regarding their MTR abundance, *Glycolysis IV* and *Glycolysis III* showed the highest abundance from key pathways. At the same time, *GDP-mannose biosynthesis*, *Purine nucleobases degradation, Glyoxylate cycle*, and *Glycogen biosynthesis I (from ADP-D-glucose)* were the least abundant key pathways all with a contribution below 1%. **Supplementary Figure 1** shows all key pathways in metabolic diagrams. **Supplementary Figure 2** shows MTR vs MG abundance of critical pathways and their corresponding correlation coefficients according to Spearman’s.

### Multiple bacterial taxa contribute to key pathways associated with COPD status

**Figure 3:**
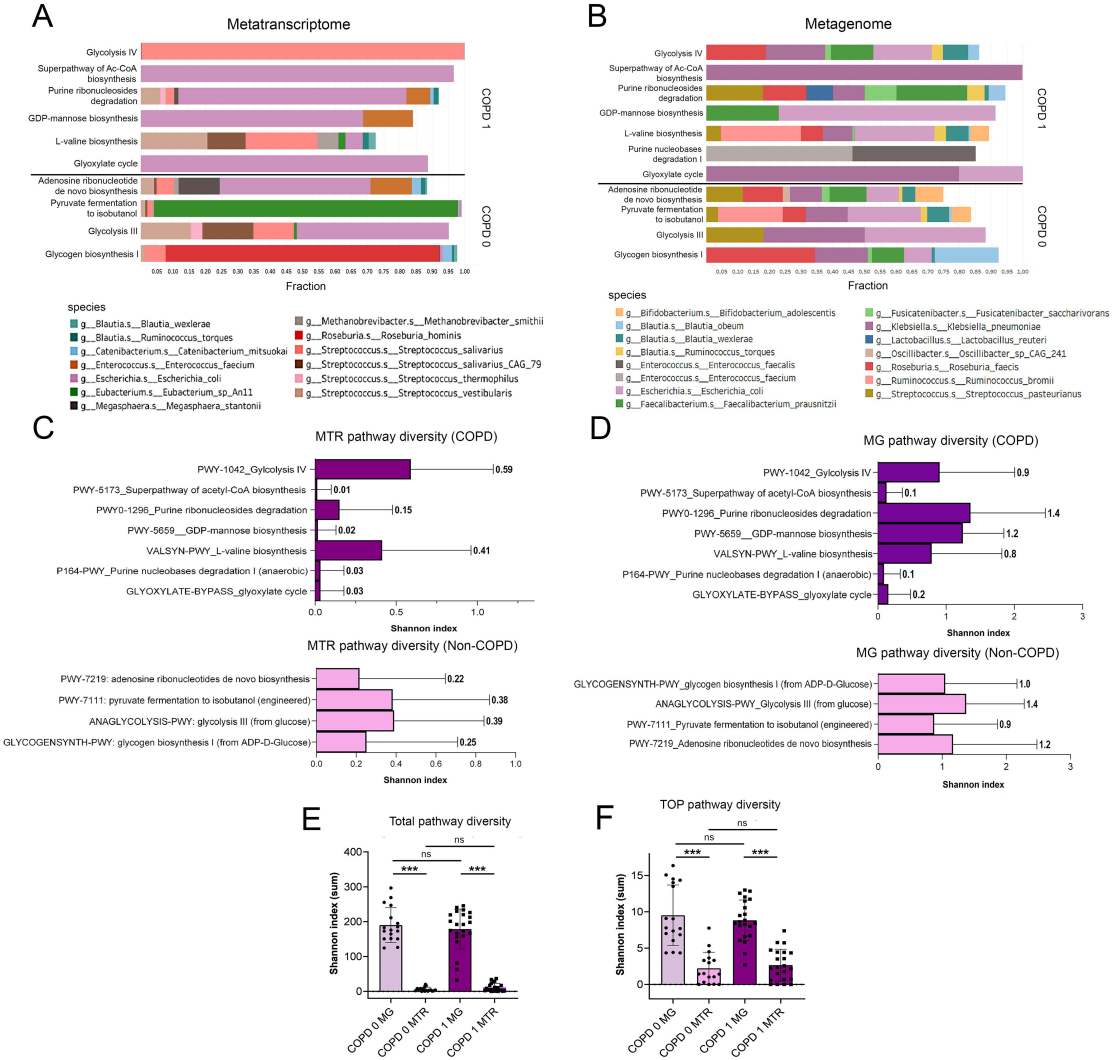
Taxonomic break-down of key pathways and pathway diversity. When assessing the taxonomic contribution of key pathways in COPD, taxa with ≥0.1% of total MTR and ≥1% of total MG abundance were analyzed **(A, B)**. *Escherichia coli* was predominant in most MTR pathways, except for *Glycolysis IV and L-valine biosynthesis*, where *Streptococcus* species (*S. salivarius, S. vestibularis*) were dominant. For non-COPD pathways like *Pyruvate fermentation to isobutanol* and *Glycogen biosynthesis I, Eubacterium_sp_An11* and *Roseburia hominis* were key contributors **(A)**. MG abundance showed greater diversity with species including *Klebsiella pneumoniae, Roseburia faecis*, and *Bifidobacterium adolescentis* contributing significantly, despite their limited role in MTR pathways **(B)**. *Purine nucleobases degradation I* pathway had no identified taxonomic contributors at MTR level. Shannon diversity indices reflecting alpha-diversity of species in each Pathway is shown on horizontal bar charts **(C, D)**. A generally higher diversity is indicated in MG than in MTR pathways both in COPD [p<0.001, total **(E)** and key pathways **(F)**] and in non-COPD patients [p<0.001, total **(E)** and key pathways **(F)**]. However, there was no significant difference in the Shannon diversity index between COPD and non-COPD patients across all analyzed pathways **(E, F)**. NS, not significant. Statistical significance ***P<.001, all p-values were two-sided.

Next, we assessed the taxonomic contribution of key COPD pathways, where the MTR and MG abundances of relevant bacterial species are displayed. Only taxa with at least 0.1% of total MTR abundance and with at least 1% of total MG abundance are shown (**Figures 3A, B**). Contributing species not reaching the minimum threshold were omitted from the stacked charts. Regarding the taxonomical composition of MTR pathways, Escherichia coli dominates in most pathways. Exceptions include COPD-specific pathways *Glycolysis IV* and *L-valine biosynthesis*, where *Streptococcus* species, including *S. salivarius* and *S. vestibularis* dominate. Regarding non-COPD specific pathways *Pyruvate fermentation to isobutanol and Glycogen biosynthesis I*, *Eubacterium_sp_An11* and *Roseburia hominis* were the strongest contributors, respectively (**Figure 3A**).

Regarding their MG abundance, taxonomical contributions were more diverse for key pathways (compared to their MTR abundance). While *E.coli* remained a significant contributor in the majority of pathways, other species such as *Klebsiella pneumoniae, Roseburia faecis, Ruminococcus bromii, Faecalibacterium prausnitzii, Streptococcus pasteurianus, Blautia wexlare, B. obeum, and Bifidobacterium adolescentis* also occurred as contributing species, whereas they were not present as important MTR contributors (**Figure 3B**). There were no identifiable taxonomic contributors in the case of *Purine nucleobases degradation I* Pathway.

Shannon diversity index was calculated to assess pathway diversity in every patient, that refers to the alpha-diversity of bacterial species contributing to each Pathway. Diversity indices for key pathways and for all pathways were calculated in COPD and non-COPD patients. In the case of key pathways, generally, MG pathway diversity was significantly higher than corresponding MTR pathway diversity, which is also reflected in the taxonomic composition (**Figures 3C, D**). Altogether, there were no significant differences in Shannon diversity index between COPD- and non-COPD patients, neither when including only key pathways nor all the analyzed pathways (both MG and MTR, **Figures 3E–F**). **Supplementary Figure 3** shows taxonomic break-down of key pathways with 0.01% (MTR, **Supplementary Figure 3A**) and 0.1% (MG, **Supplementary Figure 3B**) cut-offs regarding species contribution.

### COPD-related clinical parameters show linear correlation with key pathway-abundance

**Figure 4:**
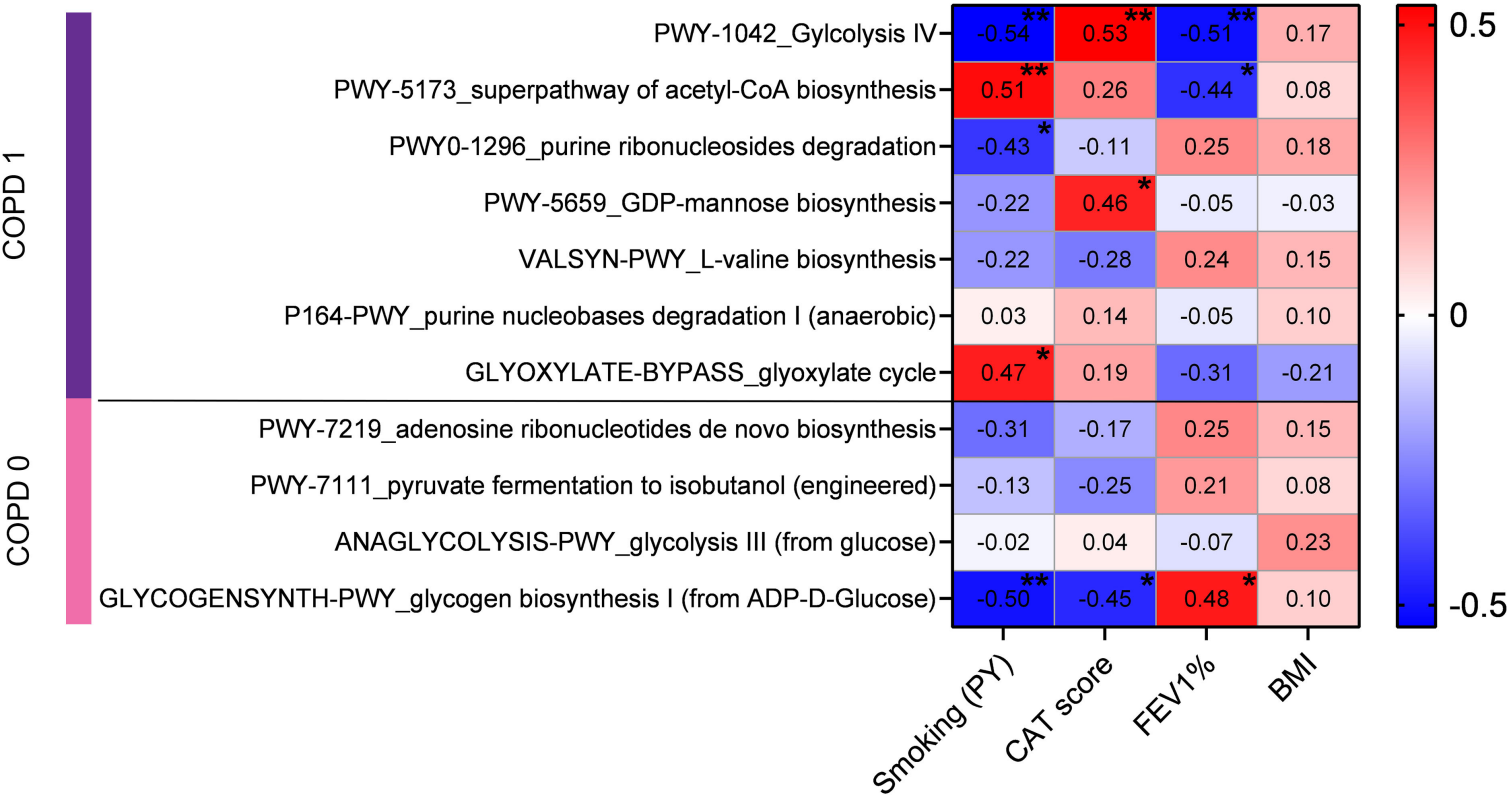
Correlation of COPD-related clinical parameters with the abundances of key pathways. Clinical parameters of patients, including FEV1%, CAT score, smoking pack-years (PY), and BMI, were correlated with the MTR abundance of key pathways using Spearman’s coefficients. COPD-specific pathway Glycolysis IV had significant negative correlations with FEV1 (rs=-0.51, p=0.009) and smoking PY (rs=-0.54, p=0.003), and a positive correlation with CAT score (rs=0.53, p=0.007). Superpathway of acetyl-CoA biosynthesis showed a negative correlation with FEV1 (rs=-0.44, p=0.028) and positive with smoking PY (rs=0.51, p=0.006). Purine ribonucleosides degradation and GDP-mannose biosynthesis were negatively correlated with smoking PY (rs=-0.43, p=0.024) and positively with CAT score (rs=0.46, p=0.024), respectively. Glyoxylate cycle was positively correlated with smoking PY (rs=0.47, p=0.011). Among non-COPD pathways, only Glycogen biosynthesis I showed significant positive correlation with FEV1 (rs=0.48, p=0.015) and negative correlations with CAT score (rs=-0.45, p=0.026) and smoking PY (rs=-0.5, p=0.007). No pathway demonstrated significant correlation with BMI. Statistical significance *P < 0.05; **P < 0.01, all p-values were two-sided.

We used the available clinical parameters of patients to correlate them with the abundance of key pathways, including FEV1%, CAT score, smoking PY and BMI. Spearman’s correlation coefficients were calculated between the MTR abundance of key pathways and value of clinical parameters (**Figure 4**). From COPD-specific pathways, Glycolysis IV showed a significant negative correlation with FEV1 (rs=-0.51) and smoking PY (rs=-0.54), and a significant positive correlation with CAT score (rs=0.53). In contrast, the *Superpathway of acetyl-CoA biosynthesis* showed a significant negative correlation with FEV1 (rs=-0.44) and a significant positive correlation with smoking PY (rs=0.51). Pathway *Purine ribonucleosides degradation* was negatively correlated with smoking PY (rs=-0.43) and pathway *GDP-mannose biosynthesis* was positively correlated with CAT score (rs=0.46). The *Glyoxylate cycle* pathway showed significant correlation with smoking PY (rs=0.47). Regarding non-COPD specific pathways, only *Glycogen biosynthesis I* showed significant correlations, including a significant positive correlation with FEV1 (rs=0.48), and significant negative correlations with CAT score (rs=-0.45) and smoking PY (rs=-0.5). No Pathway showed any kind of significant correlation with BMI (**Figure 4**).

### Metabolic SuperPathways

**Figure 5:**
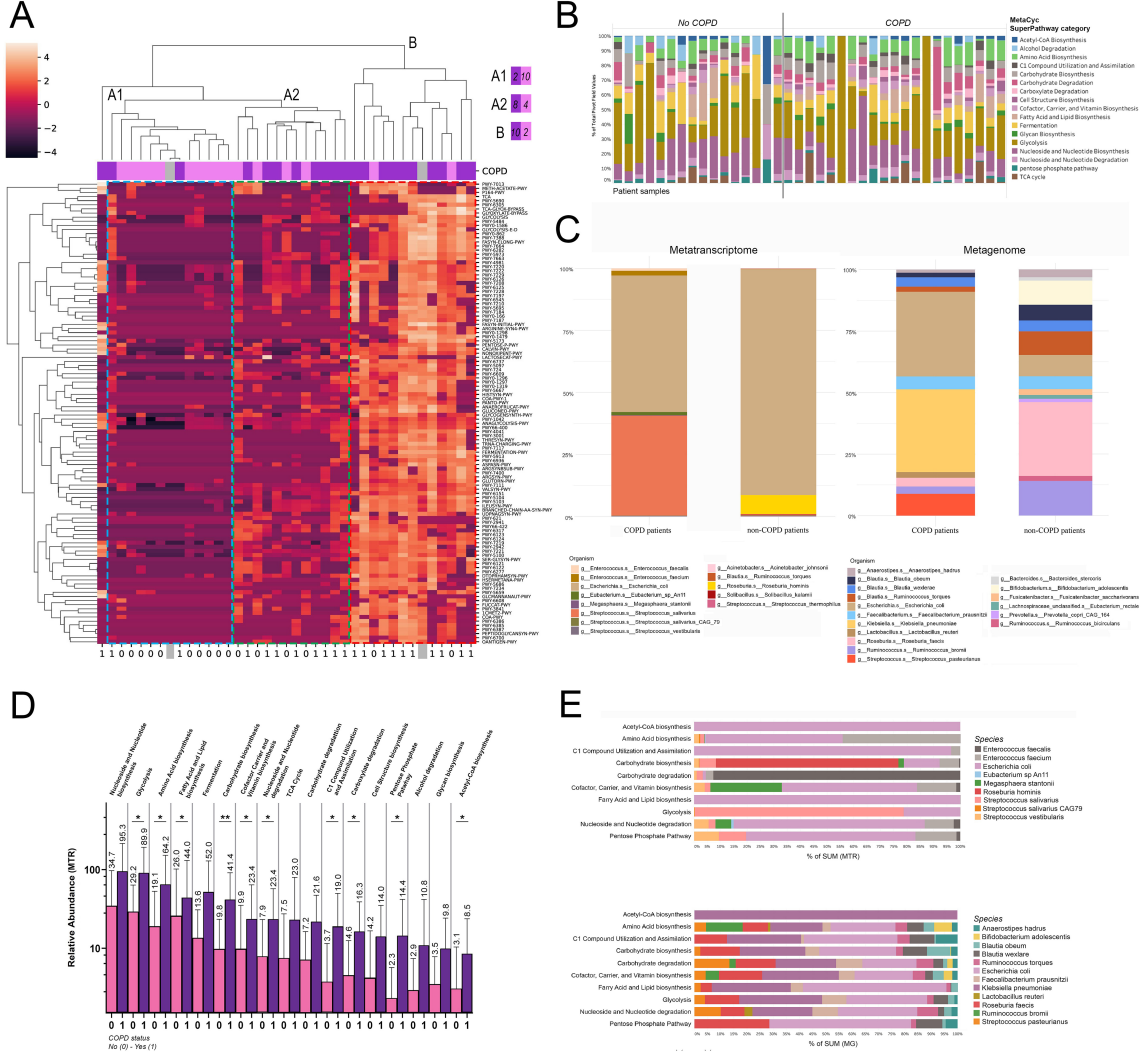
MetaCyc SuperPathways and their taxonomic composition in COPD. Metabolic pathways in patients were classified into SuperPathways, analyzed via hierarchical cluster analysis, revealing two clusters: Cluster A (subclusters A1 with low and A2 with low-to-moderate SuperPathway abundances) and Cluster B (high abundance in most SuperPathways). 83.3% of cluster B’s patients were diagnosed with COPD, compared to 16.6% in Cluster A1. One COPD patient was an outlier **(A)**. After removing SuperPathways below 1% of total contribution, 17 remained. Panel **(B)** shows their proportional distribution in all patients **(B)**. MTR and MG abundances in these pathways showed Streptococcus salivarius significantly more abundant in COPD patients (40.3% vs 0.3%) and *E.* coli in non-COPD patients (91.1% vs 55%) for MTR; diverse MG distribution with species like Streptococcus pasteurianus and Klebsiella pneumoniae more abundant in COPD **(C)**. 10 from the 17 major SuperPathways in COPD vs non-COPD patients had notably higher abundance in pathways such as Glycolysis (p=0.034), Amino Acid biosynthesis (p=0.035), Fatty Acid and Lipid biosynthesis (p=0.036), Carbohydrate biosynthesis (p=0.008), Cofactor Carrier and Vitamin Biosynthesis (p=0.048), Nucleoside and Nucleotide degradation (p=0.03), C1 Compound Utilization and Assimilation (p=0.016), Carboxylate degradation (p=0.04), Pentose Phosphate Pathway (p=0.04), and Acetyl-CoA biosynthesis (p=0.047) in COPD patients **(D)**. Taxonomic analysis showed E coli dominating many pathways, while others like Carbohydrate biosynthesis were led by Roseburia hominis, when analyzing MTR E. Enterococcus (E.) faecium notably contributed to 7 of the 10 SuperPathways, whereas E faecalis dominated SuperPathway Carbohydrate degradation. MG abundances presented a balanced contribution from various species E. A comparison of MG and MTR abundances highlighted E coli’s higher transcriptomic activity, contrasting K. pneumoniae’s strong metagenomic presence with minimal transcriptomic activity. Only taxa contributing to at least 0.1% of total MTR abundance, and 1% of total MG abundance are displayed in panels **(B, C, E)**. Statistical significance *P < 0.05; **P < 0.01, all p-values were two-sided.

We classified metabolic pathways to SuperPathway categories according to the iteration of the BioCyc platform and evaluated SuperPathway composition clustered in all patients (**Figure 5A**). Hierarchical cluster analysis with complete linkage was used to assess the grouping of patients according to their SuperPathway composition, where two major clusters emerged: cluster A, with an A1 and A2 subcluster harbouring low abundance for most SuperPathways (cluster A1), or a range from low- to moderate abundances in distinct SuperPathway clusters (axis Y); and cluster B, with a generally high abundance for the majority of identified SuperPathways. While in cluster A1 only 16.6% of patients are with COPD-comorbidity, in cluster B, 83.3% of patients are diagnosed with COPD. Cluster A1 represents an intermediate group with more balanced distribution between the two patient groups (66.7% COPD, 33.3% non-COPD). One patient with COPD was an outlier regarding its SuperPathway composition and did not belong to any of the identified clusters.

After the removal of low-abundance SuperPathways (below 1% to total contribution), 17 major SuperPathways remained (**Figure 5B**), of whom we evaluated the taxonomic composition in COPD and non-COPD patients using MTR and MG abundances (**Figure 5C**). Regarding MTR, Streptococcus salivarius was more abundant by orders of magnitude in COPD patients compared to non-COPD patients (40.3% vs 0.3%). In contrast, the E.coli was more represented in patients without COPD comorbidity (91.1% vs 55%). Enterococcus (E) faecalis and E. faecium occurred only in COPD patients above the 1% threshold (1% and 1.9%). However, Roseburia hominis RNA was only relevant in non-COPD patients (7.6%). Regarding MG, the taxonomic distribution was more diverse in both patient groups. Major differences include Streptococcus pasteurianus (8.9% vs below threshold), E. coli (34.4% vs 8.6%) and Klebsiella pneumoniae (33.6% vs below threshold) being more abundant in COPD patients (compared to non-COPD); whereas Ruminococcus bromii (14% vs 3%), Roseburia faecis (30% vs 3.5%) and Bifidobacterium adolescentis (9.8% vs below threshold) being more abundant in patient without COPD.

Next, we explored the MTR abundance of the 17 major SuperPathways in COPD vs non-COPD patients (**Figure 5D**), where multiple SuperPathways including *Glycolysis, Amino acid biosynthesis, Fatty acid and lipid biosynthesis, Carbohydrate biosynthesis, Nucleoside and nucleotide degradation, Cofactor carrier and vitamin biosynthesis, C1 compound utilization and assimilation, Carboxylate degradation, Pentose phosphate pathway* and *Acetyl-CoA biosynthesis* showed significantly increased abundance in COPD patients (compared to non-COPD patients, **Figure 5D**).

Moreover, we evaluated the taxonomic composition of SuperPathways that showed differential abundance in COPD patients, based on the same methodology as in the case of key pathways. Regarding the taxonomical composition of MTR pathways, E. coli dominates multiple SuperPathways, such as *Acetyl-CoA biosynthesis*, *C1 compound utilization and assimilation, Fatty acid and lipid biosynthesis, Nucleoside and nucleotide degradation* and *Pentose phosphate pathway.* However *Carbohydrate biosynthesis* is dominated by *Roseburia hominis, Carbohydrate degradation* is dominated by *Enterococcus faecalis* and *Glycolysis* is dominated by *Streptococcus salivarius.* Among other species *Enterococcus faecium* and *Streptococci*, including *S. vestibularis* also contribute notably to major COPD-related SuperPathways (**Figure 5E**). In the case of MG abundances, *Klebsiella pneumoniae* and *E. coli* dominate approximately equally most of the SuperPatways, but *Roseburia faecis*, *Streptococcus pasteurianus*, *Ruminococcus bromii*, *Faecalobacterium prausnitzii* and *Blautia obeum* and *wexlare* are also important contributors (**Figure 5E**). Analysing separately the contribution of species to MG and MTR abundance revealed striking divergences. E. coli seems to represent a much stronger transcriptomic activity than its MG abundance indicates, whereas K. pneumoniae features strong metagenomic presence in most of the pathways, but with virtually no transcriptomic activity. **Supplementary Figure 4** shows taxonomic break-down of top SuperPathways with 0.01% (MTR, **Supplementary Figure 4A**) and 0.1% (MG, **Supplementary Figure 4B**) cut-offs regarding species contribution.

Next, we performed Spearman’s correlation with the same clinical parameters as in the case of key pathways. The value of FEV1 showed significant correlation with SuperPatways Carbohydrate degradation (rs=-0.431), Glycan biosynthesis (rs=-0.49) and Glycolysis (rs=-0.33). Linear regression confirmed the significant interrelation in the case of Carbohydrate degradation (p=0.004) and Glycan biosynthesis (p=0.003) but not in the case of Glycolysis (p=0.0096). CAT score, Smoking PY and BMI showed no significant association with any of the SuperPathways.

### Protein domain families

**Figure 6:**
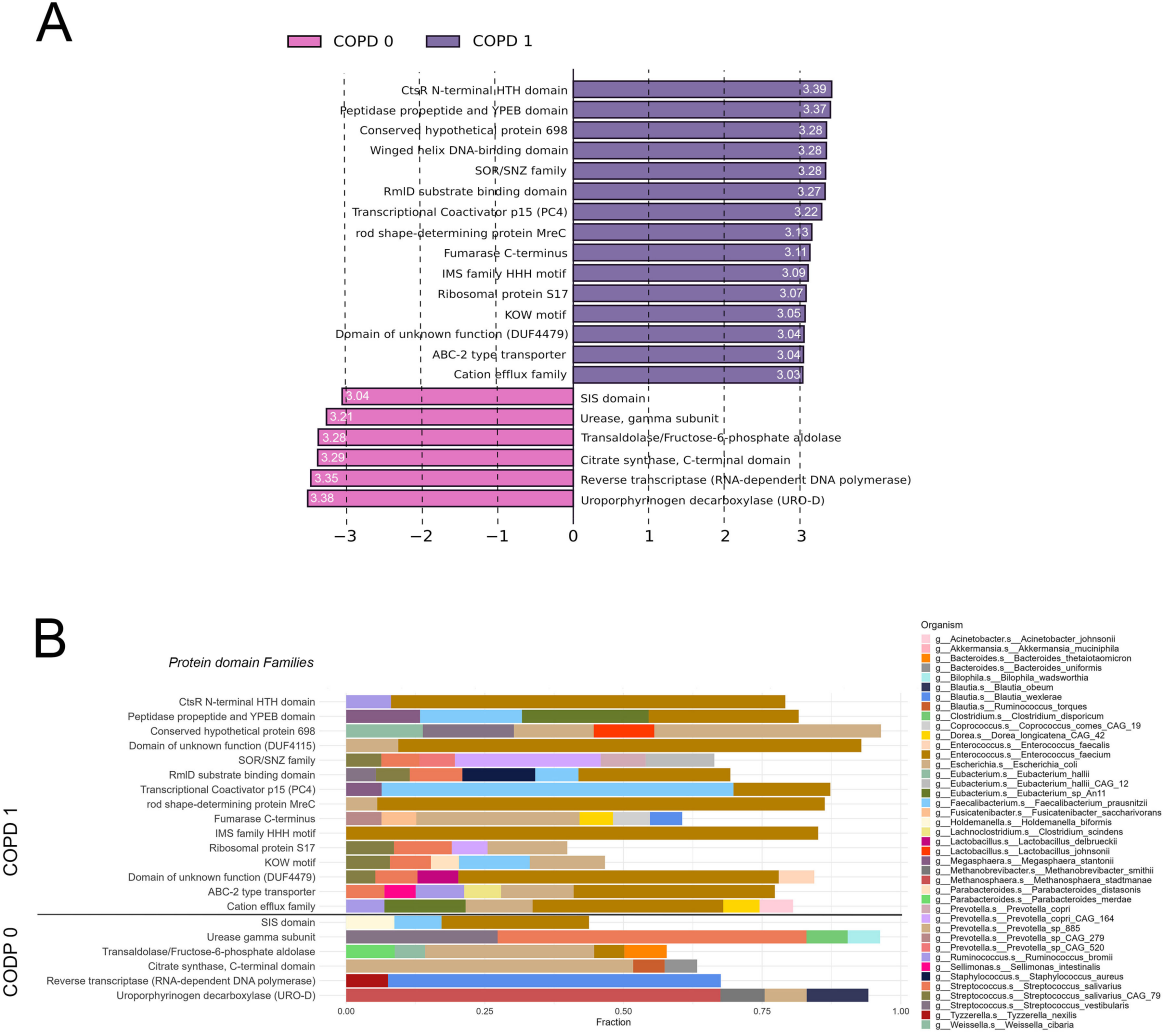
Protein domain families associated with COPD according to the gut metatranscriptome. Linear Discriminant Analysis Effect Size (LEfSe) identified 21 key PFAMs significantly discriminating between COPD and non-COPD patients based on MTR abundance (FDR<0.05, _Log10_LDAcoeff>3). _Log10_LDAcoeff values are displayed on horizontal bars showing PFAMs. Of the analyzed PFAMs, 15 showed a more significant effect size toward the COPD phenotype, while 6 PFAMs were more associated with the non-COPD phenotype **(A)**. Taxonomic analysis at the MTR level showed Enterococcus faecium dominating in 10 COPD-related PFAMs, E.coli in 8, and Streptococcus salivarius (including S. salivarius CAG79) in 6, while Faecalobacterium prausnitzi appeared in 4. For the non-COPD-related PFAMs, Blautia wexlerae and Ruminococcus torques were prominent, along with E. coli contributing to 3 PFAMs. Only taxa contributing to at least 5% of total MTR abundance are displayed in panel **(B)**.

LEfSe analysis was performed to determine key protein domain families (PFAMs) exhibiting the greatest effect size discriminating COPD vs. Non-COPD patients regarding their metatranscriptomic abundance. A total of 21 PFAMs showed statistically significant discriminating power (FDR<0.05), setting the cutoff for _Log10_LDAcoeff ≥ 3. 15 of these PFAMs showed greater effect size towards the COPD-phenotype, including *CtsR N-terminal HTH domain*, *Peptidase propeptide and YPEB domain*, *Conserved hypothetical protein 698*, *Winged helix DNA-binding domain*, and *SOR/SNZ family* among the top 5; and 6 PFAMs towards the non-COPD phenotype, including *Uroporphyrinogen decarboxylase*, *Reverse transcriptase*, *Citrate synthase*, *Fructose-6-phosphate aldolase* and *Urease gamma subunit* among the top 5 (**Figure 6A**). Taxonomic breakdown reveals a strong contribution from Enterococcus faecium (10 PFAMs), E.coli (8 PFAMs), Streptococcus (S) salivarius and S. salivarius CAG79 (6 PFAMs in total), and Faecalobacterium prausnitzi (4 PFAMs) at the MTR level in COPD-related PFAMs. In contrast, in the 6 non-COPD-related PFAMs, species such as Blautia wexlerae (*Reverse transcriptase*) and Ruminococcus torques (PFAM *Uroporphyrinogen decarboxylase*) dominate apart from E. coli (3 PFAMs). **Supplementary Figure 5** shows PFAM MTR taxonomic contributions with 1% cutoff and MG taxonomic contributions with 1% and 5% cut-offs.

## Discussion

Changes in the human gut microbiome have been linked to a variety of chronic diseases, including obesity, IBD, type 2 diabetes, cancer, cardiovascular disease, and COPD (Shreiner et al., 2015; Bowerman et al., 2020). A substantial amount of research has shown the metagenomic potential of fecal communities, including predicting anti-PD1 immunotherapy efficacy and toxicity (Human Microbiome Project Consortium, 2012; Le Chatelier et al., 2013; Limeta et al., 2020; Dora et al., 2023b; Dora et al., 2023a), but less is known about the microbiome’s transcriptional activity. The metatranscriptome represents a connection between the metagenome and community phenotype, and understanding its functional ecology requires the characterization of contributing metabolic pathways. Here, in our study, we revealed that specific bacterial phyla are present with a higher MTR abundance in the gut, than expected based on their MG abundance, including Actinobacteria, Proteobacteria, Verrucomicrobia and the Archaea Euryarchaeota. In contrast, Bacteroidetes seem to contribute lower to the gut’s MTR activity, than its Metagenomic abundance suggests. Among others, Collinsella, Streptococcus, Escherichia, Enterococcus are the most transcriptionally active genera, reflected in Pathways and Superpathways’ taxonomic representation.

When analysing data on COPD comorbidity, we find that patients with COPD exhibit a transcriptionally more active gut microbiome with increased abundance detected in most major metabolic pathways. Furthermore, the taxonomic diversity of metatranscriptomic pathways is considerably lower compared to metagenomic pathways with lesser species showing real-life transcriptomic activity despite the presence of their DNA in analysed samples. In contrast, species such as S. salivarius, S. vestibularis, E. faecalis and E. faecium were shown to be transcriptionally active, with low metagenomic abundance present in key COPD-related pathways. Finally we showed that certain pathways significantly correlate with physiological parameters frequently evaluated in COPD patients, including smoking pack year, CAT-score and FEV1.

Among key pathways, two different forms of Glycolysis show differential abundance according to COPD-comorbidity. Glycolysis IV, overabundant in COPD-patients and metatranscriptionally represented mainly by Streptococci, uses sucrose as a direct source, whereas non-COPD associated metabolic pathway Glycolysis III starts with glucose and represented by more bacterial taxa, including E. coli and E. faecalis, Megasphaera stantonii and S. salivarius. This might implicate a divergence in the anaerobic energy-homeostasis of commensal gut bacteria in the case of chronic lung inflammation (Bhayani et al., 2022). Interestingly, key pathway Glycolysis IV, apart from being positively- and negatively associated with CAT score and FEV1, respectively, it showed a moderate negative correlation with Smoking PY. This finding might seem controversial unbeknownst about 30% of COPD patients are non-smokers according to the multicenter canCOLD epidemiological study (Tan et al., 2015). Thus, Streptococci-driven glycolysis might indicate the existence of a smoking-independent pathophysiological link through the gut-lung axis. Of note, these results based on pure correlation are utmost hypothetical and need rigorous experimental validation. Superpathway of Ac-CoA Synthesis and Glyoxylate cycle, in contrast, showed significant positive correlation with pack year, implicating them in the pathogenesis of smoking-associated COPD. Unlike the citric acid cycle which is geared towards energy production, the glyoxylate cycle specializes in the biosynthesis of carbohydrates from fatty acids. This adaptation allows organisms to convert acetyl-CoA, derived from fatty acids’ breakdown into glucose. In the complex environment of the gut, where nutrients fluctuate, the glyoxylate cycle provides bacteria with a flexible metabolic pathway to utilize fats and oils, potentially derived from the diet, to synthesize glucose. This supports bacterial growth and survival and influences the gut’s overall health and function by impacting the microbial composition and metabolic outputs (Proffitt et al., 2022). Recent study demonstrated the glyoxylate cycle’s key role in maintaining metabolic balance and stress resistance using a viable, but nonculturable bacteria (VBNC) model (Qi et al., 2023). Glycogen Biosynthesis I (from ADP-D-glucose) was the only Pathway that showed consistently a positive correlation with lung function (CAT score and FEV1) and negative correlation with smoking. Intracellular glycogen accumulation in several gut commensals acts as a niche adaptation trait, aiding in the colonization and adaptation to the gastrointestinal tract, and enhancing survival in the competitive and dynamic gut ecosystem​ (Esteban-Torres et al., 2023).

Comprehensive gut microbiome analysis identified multiple Streptococcus species, including *S.Salivarius* and *S. parasanguinis* to be overrepresented in COPD patients and to correlate with reduced lung function (Bowerman et al., 2020). Furthermore, the latter taxa contribute to a COPD-associated metabolic network that is associated by pulmonary inflammation (Bowerman et al., 2020). Others reported Prevotellaceae as a significantly more abundant family in mild COPD patients (compared to healthy), and showed a trend for Ruminococcaceae and Enterococcaceae being overrepresented in GOLD III-IV COPD compared to healthy controls (Li N. et al., 2021). Altogether both studies recapitulated the fact that gut microbiome is not altered considerably at a taxonomic level, but rather changes in its functionality and metabolomics (Bowerman et al., 2020; Li N. et al., 2021). Regarding metatranscriptomics, our current study showed that multiple Streptococcus species dominate pathways overrepresented in COPD, especially Glycolysis IV. Interestingly, our data suggests that E. coli is a major contributor at a metatranscriptomic level to all relevant pathways regardless of COPD, but not at a metagenomic level. The Carbohydrate Biosynthesis superpathway was dominated by Roseburia hominis metatranscriptionally. Roseburia species are known for their butyrate-producing capability, a short-chain fatty acid essential for colonic health and possessing anti-inflammatory properties (Louis and Flint, 2009).

Enterococcus faecalis and faecium occur as an important contributor both in key metabolic pathways and major superpathways at a metatranscriptomic level, but its abundance is not noticeable if we observe the metagenom, the difference is even more dominant in COPD patients. This suggests a discrepancy between the abundance and the transcriptomic activity of these bacteria in the gut. Concerning metabolic pathways, metatranscriptomic diversity is significantly lower than metagenomic diversity, implicating a form of convergence, where only a fraction of species are active regarding their gene expression. When observing metabolism at a macro level, a general increase in transcriptomic activity occurs in COPD, with most of the SuperPathways being overexpressed. Also, in COPD patients, the taxonomic representation of major SuperPathways changes abruptly, where Streptococci (S.), including S. salivarius contributes to ~41% of total pathway abundance compared to ~1% in non-COPD patients. Previously, both Bowerman et al (Bowerman et al., 2020) and Li et al (Li N. et al., 2021), described a significant increase in Streptococci regarding their metagenomic- and metabolite abundance in COPD, but no data was presented at the transcriptomic level. Also, the metagenomic presence of E. coli is more dominant in COPD patients, that is in line with Bowerman et al (Bowerman et al., 2020), but not transcriptionally, where E.coli represents a relatively smaller fraction of abundance due to the dominance of Streptococci. In contrast, Roseburia faecis and Ruminococcus bromii showed an increased presence in patients without COPD comorbidity, corresponding to earlier findings in the field (Bowerman et al., 2020; Li N. et al., 2021). Interestingly, Klebsiella pneumoniae was overrepresented in COPD patients at the metagenomic level. However, Klebsiella species appear to be transcriptionally silent, not contributing significantly to any superpathway or patient group. Plus, Bifidobacterium adolescentis genome showed increased abundance in non-COPD patients, but showed no significant presence in the metatranscriptome. Neither Klebsiella pneumoniae nor Bifidobacterium adolescentis in the gut was described earlier in connection with COPD.

Protein domain families (PFAMs) are families of protein domains or conserved protein sequences. Identifying Pfams in the metagenomic or metatranscriptomic data is done by employing Hidden Markov Models (HMMs) to search for known protein domains within the sequence data. Here, we showed that multiple PFAMs were associated with COPD, including *CtsR N-terminal HTH domain*, *Peptidase propeptide and YPEB domain, or Winged helix DNA-binding domain;* and multiple PFAMs are overrepresented in patients without COPD comorbidity such as *Uroporphyrinogen decarboxylase*, *Reverse transcriptase*, or *Citrate synthase.* The CtsR regulon includes the clpC, clpP, and clpE genes, which are negatively regulated by the CtsR of L. monocytogenes, a member of the family comprising several Firmicute transcriptional repressors of class III stress genes (CtsR) implicating a role in heat-shock protein-mediated anti-stress response (Nair et al., 2000). Peptidase propeptide and YPEB domain likely has a protease inhibitory function (Yeats et al., 2004), whereas non-COPD-associated Uroporphyrinogen decarboxylase (UROD), a branch point enzyme in the biosynthesis of tetrapyrroles, catalyzes the decarboxylation of four acetate groups of uroporphyrinogen III, resulting in coproporphyrinogen III playing an essential role in the biosynthesis of heme and chlorophyll, a protein family already characterized in yeasts and Bacillus subtilis (Garey et al., 1992; Hansson and Hederstedt, 1992). Regarding Citrate synthase in bacteria, its role was identified in metabolism and bacterial cell cycle control, independent of its metabolic activity (Bergé et al., 2020). The taxonomic composition of key PFAMs garnered from MTR data corresponds with our findings from the metabolic pathway analysis, where Streptococci, E. coli, and Enterococcus faecium were the strongest contributors in COPD, and Ruminococcus torques and Blautia welfare in non-COPD. It is important to acknowledge microbial communities’ inherent dynamism and context-specific nature, highlighting that specific PFAMs may not consistently correspond to particular functions or taxa across varied environments. Consequently, the interpretations presented here primarily serve as a foundation for hypothesis generation, necessitating rigorous validation through experimentation in diverse settings.

An important confounder in microbiome research, cigarette smoking is known to reduce microbiome diversity across the body, particularly in the respiratory and GI tracts, as evidenced by studies such as Gui et al (Gui et al., 2021). and Shapiro et al (Shapiro et al., 2022). In COPD and lung cancer patients, studies show that smoking alters gut bacterial abundance, decreasing Firmicutes and Proteobacteria, while increasing Prevotella, Bacteroides, and Bacteroidetes (Ding et al., 2021; Chen et al., 2024). Shanahan et al. found higher Streptococcus and Veillonella spp. in smokers (Shanahan et al., 2018). However, few studies have compared the gut microbiotas of smoking vs. non-smoking COPD patients, and functional metagenomic studies are scarce. Bowerman et al (Bowerman et al., 2020). found no difference between these groups and due to the low number of non-smoking COPD patients in our cohort, we cannot draw solid consequences of the functional microbiome in non-smoker COPD.

Case-control studies with the recruitment of healthy, usually young participants have the setback of non-uniformity regarding age and performance status that can significantly influence the baseline microbiome (Badal et al., 2020; Ghosh et al., 2022). Our cohort includes a group of patients from the same geographic region, with similar health status and age distribution and with a comparable burden of chronic conditions that can act as confounders. Our study has limitations. The size of the population cohort is modest, and we cannot tell whether the alteration of bacterial transcriptomic activity in the gut is the cause or a consequence of chronic inflammation in the lung. Our study did not classify patients according to COPD treatment, so we cannot assess inhaled or systemic steroid therapy’s influence on the gut metatranscriptome. Metatranscriptomic analyses have the downside of increased degradability of RNA compared to DNA, which can be managed by precise quality control. Furthermore, it is important to acknowledge that transcriptome data alone may not fully capture the metabolic changes occurring in the host and often shows poor correlation with both proteomic and metabolomic profiles (Taniguchi et al., 2010; Ma et al., 2019; Li L. et al., 2021). Future research should integrate metagenomic, metatranscriptomic, and metabolomic data to provide a more comprehensive understanding of microbial community physiology and its impact on lung cancer pathology (Cavill et al., 2016). Also, a potential bias exists in public databases favoring E. coli over other Gram-negative bacteria, which may have influenced our pathway analyses. However, E. coli’s metabolic versatility and the inclusion of well-described taxa such as Bacteroides, Prevotella, and Ruminococcus in current databases suggest that our findings may still accurately reflect biological reality. Future research should validate these findings with a more diverse range of bacterial species to mitigate this bias. The administration of probiotics and prebiotics, like Bifidobacterium strains, has shown efficacy in restoring gut and lung microbiomes in diseases linked to the gut-lung axis, such as COVID-19, asthma, and COPD (Budden et al., 2017; Li et al., 2024). Dietary interventions, including foods rich in fiber, are also useful therapeutic strategies for diseases similar to COPD, due to the short-chain fatty acids produced by beneficial bacteria (Vaughan et al., 2019; Ding et al., 2021). Targeting overrepresented Streptococcus species and boosting beneficial taxa like Roseburia hominis may reduce COPD-related inflammation. Existing microbiome-targeted therapeutics for dysbiosis in IBD and metabolic diseases might offer a basis for developing microbiome-based COPD interventions.

## Conclusion

Our metatranscriptomic analysis elucidates distinct transcriptional activity within the gut microbiome of NSCLC patients, shedding light on its potential therapeutic implications in COPD comorbidity. Altogether, our findings confirmed the previously reported increased metagenomic abundance of intestinal Streptococci and E. coli in COPD at the transcriptomic level. Furthermore, we demonstrated the association of multiple metabolic pathways and protein domain families with COPD presence, suggesting a multifaceted microbiome involvement in the disease’s pathology. These findings underscore the importance of incorporating metatranscriptomic perspectives to unravel the intricate microbial interactions and their influence on chronic diseases, paving the way for novel microbiome-targeted therapeutic strategies in COPD.

## Data Availability

The data presented in the study are deposited in Figshare, accessible through the following link: https://doi.org/10.6084/m9.figshare.27633195.v1.
