# Supplementary material for: Metabolic pathways from the gut metatranscriptome are associated with COPD and respiratory function in lung cancer patients
Source: Front Cell Infect Microbiol. 2024 Nov 20;14:1381170. doi: 10.3389/fcimb.2024.1381170 (PMC11616033; doi:10.3389/fcimb.2024.1381170)
Supplement: Supplementary file 1 [file DataSheet1.docx]

**Supplementary Material**





**Supplementary Figure 1. Diagrams of key metabolic pathways associated with the presence- and the lack of COPD comorbidity in lung cancer patients.**


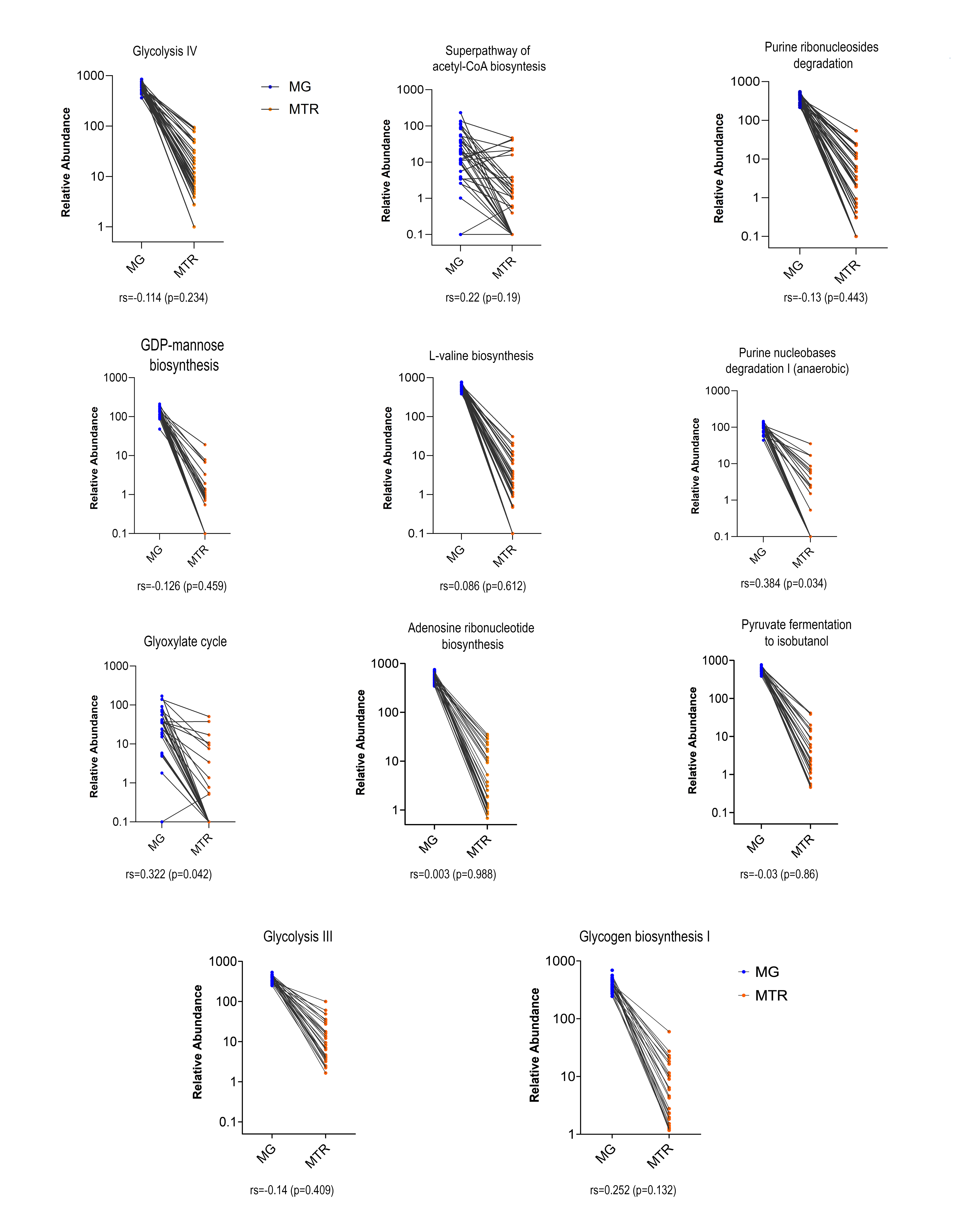


**Supplementary Figure 2. Pairwise comparison of Metagenomic (MG) and Metatranscriptomic (MTR) abundance of key pathways associated with COPD- and non-COPD phenotypes.** Relative abundances is displayed in logarithmic Y axis. Correlation coefficients are shown below charts according to Spearman’s with corresponding p-value.


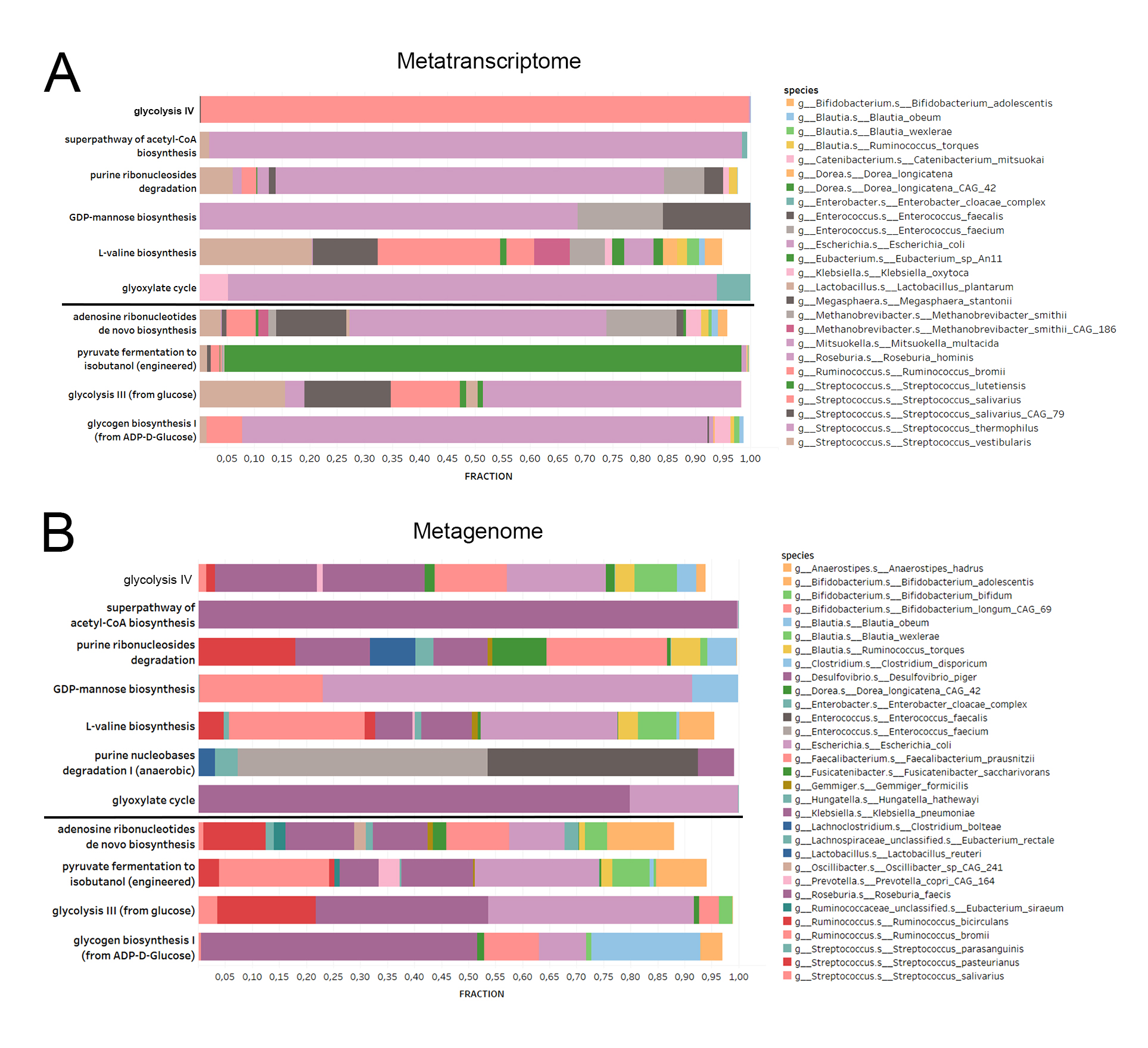


**Supplementary Figure 3. Taxonomic composition of key pathways with 0.01% (MTR, A) and 0.1% (MG, B) cut-offs regarding species contribution.** Species are displayed if they contribute to a minimum of 0.01% (MTR) or 0.1% (MG) of the corresponding pathway’s abundance.


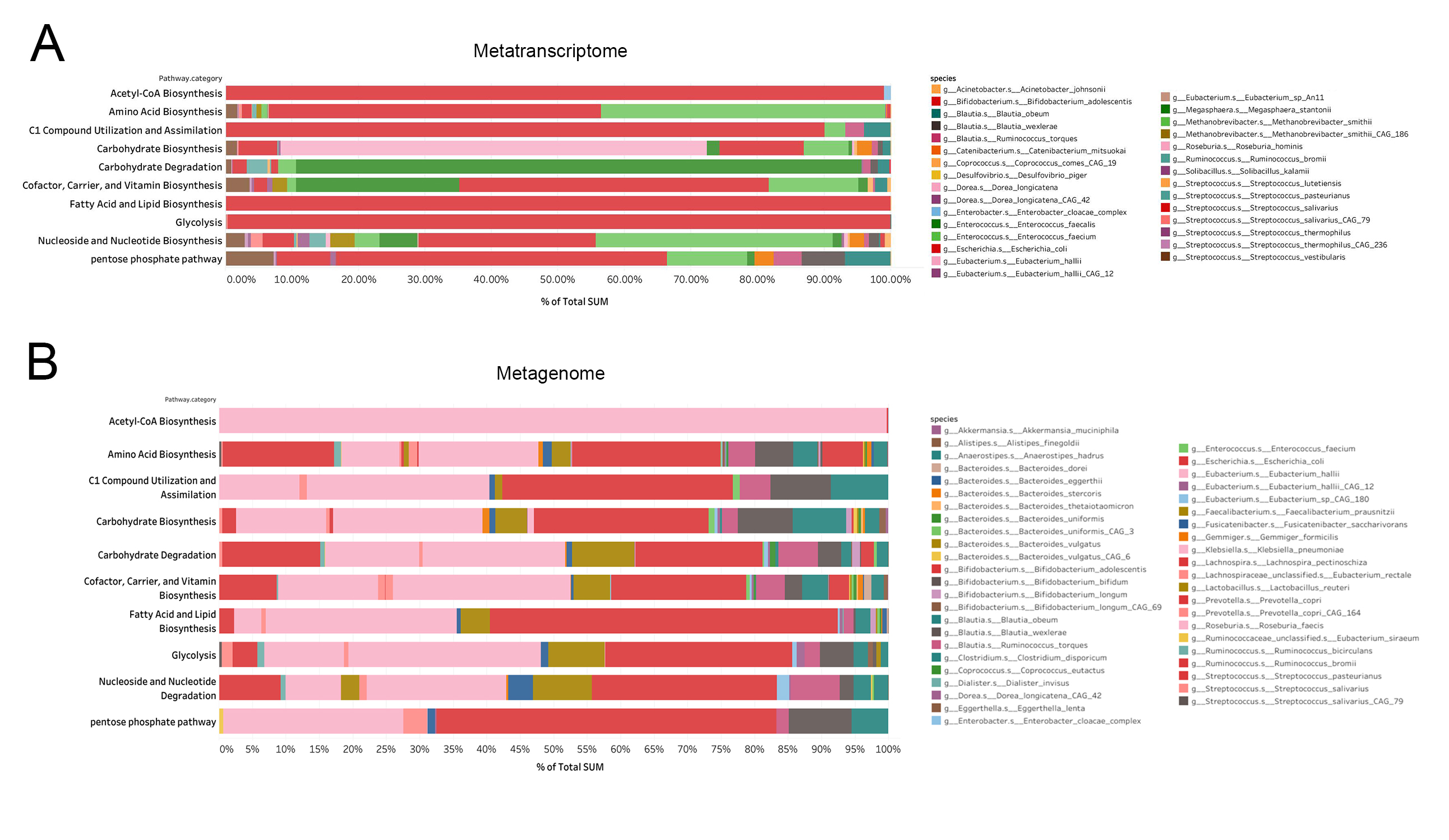


**Supplementary Figure 4. Taxonomic composition of Superpathways overrepresented in COPD with 0.01% (MTR, A) and 0.1% (MG, B) cut-offs regarding species contribution.** Species are displayed if they contribute to a minimum of 0.01% (MTR) or 0.1% (MG) of the corresponding pathway’s abundance.


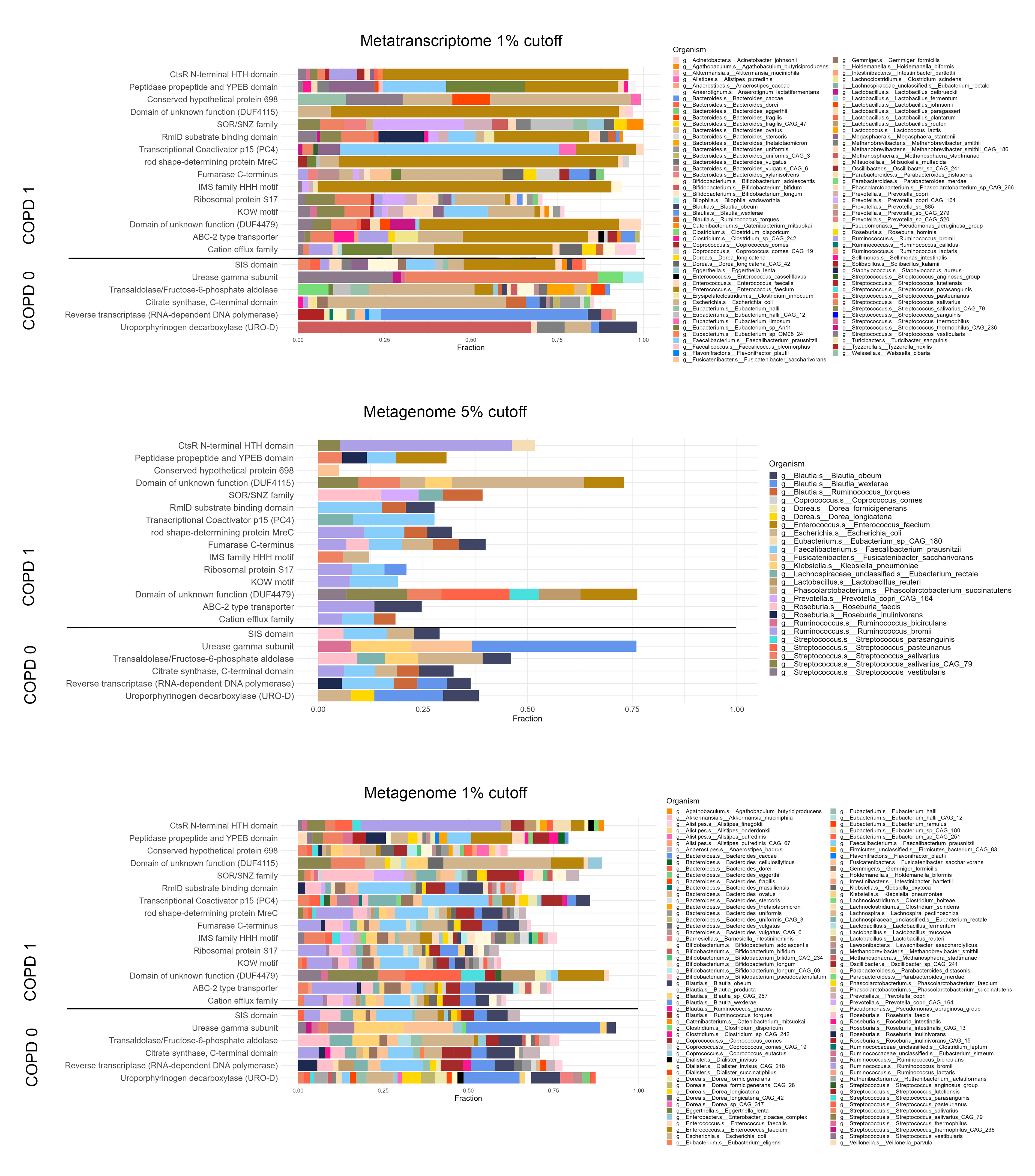


**Supplementary Figure 5. Taxonomic composition of key protein domain families (PFAMs) with 1% species contribution cutoff regarding their MTR abundance (A), and with 5% (B) and 1% cut-offs (C) regarding their MG abundance.**
